# Evaluation of the Cost Effectiveness of Vesico-Amniotic Shunting in the Management of Congenital Lower Urinary Tract Obstruction (Based on Data from the PLUTO Trial)

**DOI:** 10.1371/journal.pone.0082564

**Published:** 2013-12-20

**Authors:** Lavanya Diwakar, Rachel K. Morris, Pelham Barton, Lee J. Middleton, Mark D. Kilby, Tracy E. Roberts

**Affiliations:** 1 Health Economics Unit, School of Health and Population Sciences, The Public Health Building, University of Birmingham, Birmingham, United Kingdom; 2 School of Clinical and Experimental Medicine, College of Medical and Dental Sciences, University of Birmingham, Birmingham, United Kingdom; 3 Birmingham Clinical Trials Unit, University of Birmingham, Birmingham, United Kingdom; 4 Fetal Medicine Centre, Birmingham Women's Hospital NHS Foundation Trust, Edgbaston, Birmingham, United Kingdom; University of Alabama at Birmingham, United States of America

## Abstract

**Objective:**

To determine the cost-effectiveness of in-utero percutaneous Vesico Amniotic Shunt (VAS) in the management of fetal lower urinary tract obstruction (LUTO)

**Design:**

Model based economic analysis using data from the randomised controlled arm of the PLUTO (percutaneous vesico-amniotic shunting for lower urinary tract obstruction) trial.

**Setting:**

Fetal medicine departments in United Kingdom, Ireland and Netherlands.

**Population or Sample:**

Pregnant women with a male, singleton fetus with LUTO.

**Methods:**

Costs and outcomes were prospectively collected in the trial; three separate base case analyses were performed using the intention to treat (ITT), per protocol and uniform prior methods. Deterministic and probabilistic sensitivity analyses were performed to explore data uncertainty.

**Main Outcome Measures:**

Survival at 28 days, 1 year and disease free survival at 1 year.

**Results:**

VAS was more expensive but appeared to result in higher rates of survival compared with conservative management in patients with LUTO. Using ITT analysis the incremental cost effectiveness ratios based on outcomes of survival at 28 days, 1 year, or 1 morbidity-free year on the VAS arm were £15,506, £15,545, and £43,932, respectively.

**Conclusions:**

VAS is a more expensive option compared to the conservative approach in the management of individuals with LUTO. Data from the RCT suggest that VAS improves neonatal survival but does not result in significant improvements in morbidity. Our analysis concludes that VAS is not likely to be cost effective in the management of these patients given the NICE (National Institute of Health and Clinical Excellence) cost threshold of £20,000 per QALY.

## Introduction

Congenital lower urinary tract (bladder outflow) obstruction (LUTO) is an uncommon condition that is generally identified in the second trimester of pregnancy using prenatal obstetric ultrasound scan. The estimated incidence is between 1∶250 to 1∶1000 pregnancies, depending on acquisition of pre and postnatal data and whether or not terminations of pregnancy related to the condition are included [1]. The condition is associated with a high prevalence of chronic renal impairment in the newborn, infancy and childhood. When associated with significantly low amniotic fluid level (or oligohydramnios) there is a considerable risk of pulmonary hypoplasia (PH). PH has an associated perinatal mortality of at least 50%, most significantly within the first week of newborn life [2], [3]. A technique known as ‘ultrasound-directed, in-utero, percutaneous Vesico Amniotic Shunting’ (VAS) bypasses the congenital urethral obstruction to potentially improve fetal outcome [4]. A systematic review of the available literature found that it was difficult to draw meaningful conclusions given the heterogeneity in the design of the published studies for interventions for this condition and revealed potential bias in the observational data [5]. Furthermore, VAS is a specialised, labour intensive technique needing multidisciplinary input. The costs associated with VAS, including the procedure and subsequent care of surviving children, are high. Its use must therefore be justified, given the limited availability of healthcare resources [6].

Currently the National Institute of Health and Care Excellence (NICE), which is a UK government funded clinical guidance issuing authority, acknowledges that definitive guidance cannot be issued for the use of VAS given the paucity of available evidence. It recommends the procedure is only performed in specialist centres with access to appropriate multidisciplinary teams once patients have been provided with clear written information. In patients for whom the procedure was considered to have a potential benefit, clinicians were encouraged to enter the patients into the PLUTO trial or the associated registry [7].

The PLUTO (Percutaneous shunting for lower urinary tract obstruction) trial [6] is the largest randomised controlled trial to date assessing the clinical and cost effectiveness of antenatal VAS in children with LUTO. Pregnant women whose babies were identified antenatally as having the condition were invited to participate in the trial. An economic evaluation was included as an integral component of the study design to take place alongside the trial.

The trial compared in-utero percutaneous VAS against the conservative or ‘wait and watch’ approach for these children. In this paper we report the economic evaluation and thus present a full assessment of the relative cost-effectiveness of VAS against conservative treatment.

An economic evaluation is a comparison of two alternative interventions in terms of costs and outcomes [8]. NICE recommends that results of economic evaluations should be expressed in terms of cost per QALY as far as possible, where QALY refers to the economic outcome being presented in terms of Quality Adjusted Life Years (QALYs) [9]. However, no validated method is available for reliable estimation of QALYs in children aged less than 2 years.

The model based economic evaluation reported in this paper took the form of a cost-effectiveness analysis which presents results in natural units based on three pre-determined outcomes (i) survival at 28 days; (ii) survival at 1 year and; (iii) clinical morbidity free survival to 1 year (MFS). That is, the analysis assessed the additional cost incurred if VAS was to produce an additional unit of one of these defined outcomes.

Model based economic evaluations have the advantage of incorporating information from a variety of sources to evaluate the costs and benefits associated with alternative policy decisions (e.g. funding or not funding VAS for treatment of LUTO in this case) while explicitly accommodating the uncertainty associated with data inputs. These results can then be presented in a clear way to the decision maker. The analysis was carried out from the perspective of the UK National Health Service (NHS), which means that only direct costs to the UK NHS were considered.

## Methods

The full details of the PLUTO trial are presented elsewhere [10]. Briefly, following an antenatal ultrasound diagnosis of lower urinary tract obstruction in a male fetus, the mother was approached for consent to participate in the PLUTO trial. The trial had a pragmatic design and recruitment into two separate study populations was allowed based on the preference of the mother or the physician. If the mother or the physician had a strong preference for one treatment over the other, the patient was registered and outcomes were noted. Randomisation of the patients into either VAS or conservative arms of the PLUTO trial was performed only when neither treatment was favoured by either the mother or the physician. This design ensured that all patients were followed up within the trial whilst not being forced to choose a treatment when they clearly preferred one option over another. However, recruitment into the randomised controlled trial was difficult and the trial had to be stopped before its intended recruitment target was reached. Difficulties in recruitment were related to a strong patient or physician preference for a particular treatment option leading to exclusion from the RCT. Also there was a higher rate of terminations of pregnancy (ToP) than anticipated. In addition, there were difficulties in recruiting international centres to the trial.

The model based economic analysis used data only from the randomised controlled arm of the PLUTO trial. The appropriate model was a decision tree model given the short time frame of the study [11]. The model was constructed and analysed using the software package TreeAge Pro 2012 (TreeAge Software Inc., Williamstown, MA, USA, 2012). The structure of the model defining the likelihood of presentation of the condition and the treatment pathways was informed by expert opinion of the trial clinicians and supplemented with data from published systematic reviews [5], [6].

**Figure 1:**
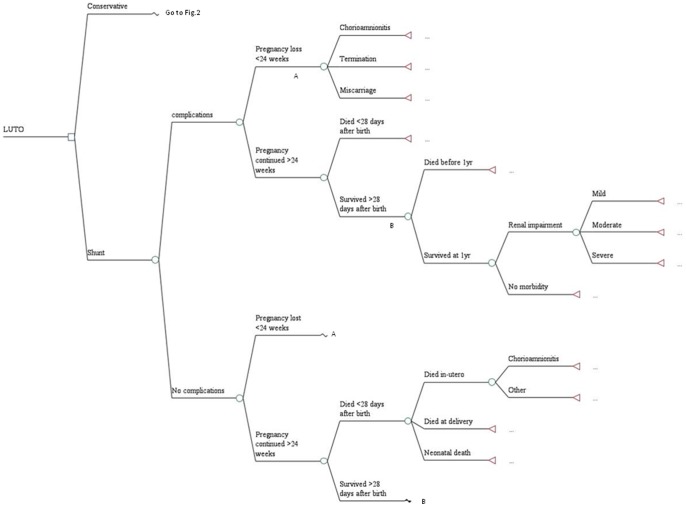
Decision tree model representing the clinical pathways experienced by patients in the VAS arm of the trial. A and B represent parts of the decision tree with the same pathways (but with different probabilities).

**Figure 2:**
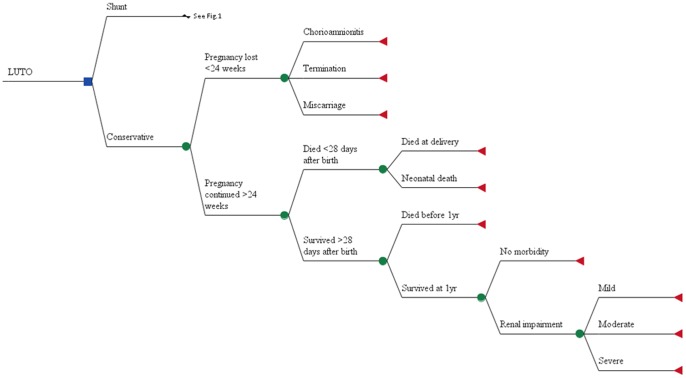
Decision tree model representing the clinical experiences of patients in the conservative arm of the trial.

In the model, we examined the antenatal, perinatal and postnatal progress of the affected fetus within the trial following randomisation to VAS or conservative arms (Figures 1 and 2). Clinical pathways experienced by the mothers until delivery and those of the children up to the first year of life within the trial are represented. A glossary of terms used in this analysis is provided in table 1.

**Table 1:** Glossary of terms used in the model.

Health state	Definition
VAS	All fetuses with LUTO (Lower Urinary Tract Obstruction) who received intra-uterine vesicoamniotic shunting
Conservative	All fetuses with LUTO who did not receive the intervention
Miscarriage	Pregnancy loss at less than 24 weeks due to unexplained causes
Died in-utero	Death of fetus due to unexplained reasons after 24 weeks of gestation
Died at delivery	died within 24 hours of birth
Neonatal death	death between 24 hrs and 28 days of birth
No morbidity	No known ongoing, chronic pathology related to LUTO or its repair
Mild renal impairment	Serum Creatinine>50 µmol/ L; No signs or symptoms of renal disease
Moderate renal impairment	Serum creatinine>50 µmol/ L; Medical management of renal disease
Severe renal impairment	Serum Creatinine>50 µmol/ L; Patient on dialysis ± Transplant being considered

Complications due to VAS placement within the trial, including chorioamnionitis (associated with spontaneous rupture of membranes) and dislodgement of shunt requiring re-insertion, were represented in the model. Probabilities for the decision tree model were derived directly from the trial data. Where only two outcomes were possible (so the data are binomial), a beta distribution was applied within the model whereas if more outcomes were possible (multinomial data), a Dirichlet distribution was used. For instance, of the 15 patients randomised to the conservative arm of the model, 3 women did not continue their pregnancy beyond 24 weeks while 12 patients continued into their third trimester (Figure 2). Since only two possibilities could occur at this node (binomial data), the probabilities were assigned a Beta distribution and were calculated as 0.2 (3/15) and 0.8 (12/15) respectively. On the other hand, a woman could experience pregnancy loss within the model at less than 24 weeks due to termination, chorioamnionitis or a miscarriage. To represent these multiple possible outcomes, a Dirichlet distribution was appropriate.

All antenatal and perinatal resource use data associated with the management of these patients including antenatal ultrasound scans, karyotyping, termination of pregnancy or miscarriage, post-mortem examination, neonatal hospital admission were collected prospectively within the trial. Data related to procedures performed, as well as resource use associated with morbidity such as renal impairment were also collected prospectively from all participating centres during the study (Table S1 in File S1). The cost for VAS placement was calculated from the ‘bottom-up’ by estimating the resource use and costing these individually (Table 2). Complications related to VAS placement such as intrauterine death or chorioamnionitis were also captured within the trial. Unit costs obtained from standard sources such as Netten and Curtis [12] and UK Healthcare Related Group (HRG) cost data [13] were applied to the data to obtain actual costs. Costs of laboratory investigations were obtained by contacting local NHS laboratories (Tables S2, S3, S4, S5 in File S1).

**Table 2:** Shunt costs.

Description	Unit Cost (£)	Source
Cost of the Vesico-amniotic shunt	£154.30	BWH*
Cost of scan	£445.50	BWH
Counselling (30 min consultant time)	£80.50	PSSRU 2011
Shunt insertion		
30 min consultant time	£80.50	PSSRU 2011
30 min assistant time (senior nurse)	£61	PSSRU 2011
Total	£821.80	

* BWH: Birmingham Women's hospital.

All costs in the model are in UK sterling in 2011 values. Health and Community Health Services (HCHS) pay and price indices were used to inflate costs, where appropriate [12]. If exact procedures were not listed in the reference costs, the closest plausible cost was inputted after discussions with the clinicians.

In order to carry out this model based economic analysis, it was necessary to make the following assumptions:

1. All centres (UK and international) were assumed to have similar expertise and protocols for management of these patients.
2. Data relating to drug use (e.g. maternal antibiotics and sedatives) were assumed to be minimal and are not included in the analysis.

### Ethics

The RCT and registry had a favourable ethical opinion from Nottingham Research Ethics Committee (MREC) approval (COREC Ref 04/Q2404/89), determining that the trial design respects the rights, safety and wellbeing of the participants. Every potential UK centre also obtained LREC (Local Research Ethics Committee) and Trust R&D approval. International centres were asked to apply for ethics committee approval according to their own systems, providing the PLUTO Trial Office with written evidence of approval.

## Analysis

Given the small number of patients within the trial, we carried out three alternative analyses for the base case in order to make the results more robust. These include i) the intention to treat (ITT) analysis, ii) per protocol analysis (excluding those patients who terminated their pregnancies following randomisation) and iii) the uniform prior analysis (a Bayesian analysis which assumes that the prior probability of each outcome is equal). The ITT analysis is discussed in detail in this paper. For the other two analyses, please refer to File S1. The probabilities for various health states represented within the model are shown in Tables S6–S8 in in File S1 attached to this article.

For all these base case analyses, deterministic and probabilistic sensitivity analyses were further carried out to assess the uncertainty associated with input parameters. Deterministic sensitivity analysis aims to estimate the effect of changing a single parameter on the overall Incremental Cost Effectiveness Ratio (ICER) obtained. The point estimates used for all the other model parameters remain unchanged. Within the current study, the following DSA were considered.

1. **Changing cost of VAS insertion (DSA1 and DSA2).** The VAS used in this trial is a specialist procedure and is not offered routinely within the NHS. Therefore, there are no HRG (Health resource group) tariffs to inform the costs of the intervention. For the purposes of base case analysis, we estimated the cost of the procedure at £821.80 by costing each of the individual components of resource use (Table 2). Since the costs of VAS placement may vary between individual centres, we investigated the effect of different VAS costs on the overall cost effectiveness of the intervention. In the absence of definite data regarding cost variability, we arbitrarily doubled and halved VAS costs in two separate sensitivity analyses.
2. **Normalise costs by adjusting extreme values (DSA3).** Within the trial, three patients had significantly higher costs than the average for the group. Assuming that this finding was totally by chance, we decided to explore the effect of omitting these outlying costs from our analysis. We, therefore, carried out a sensitivity analysis substituting these extreme costs with average costs for the group.
3. **Analysis as per treatment option delivered (DSA4).** Five individuals within the trial did not receive the treatment to which they were randomised. Whilst randomisation was respected during the base case analysis, we felt that given the small number of participants within the trial it was important to explore whether the changes to treatment in these patients (who represent about 16% of overall participants) significantly affected the overall result. Hence DSA4 considered costs and outcomes as per treatment received by the patients.

A probabilistic analysis was also carried out to explore the effects on the ICERs of the uncertainty in the model input data. Probabilistic sensitivity analysis uses random numbers to generate multiple possible estimates of cost and outcomes from within the probability distributions (i.e., the Monte Carlo principle). 20,000 simulations will be carried out using the Monte Carlo principle. The distribution around each parameter is specified by its baseline estimate and a bound (upper or lower) of the estimated 95% confidence interval. The advantage of this method is that all parameter uncertainties can be incorporated simultaneously into the analysis.

The result of this analysis will be used to obtain a cost-effectiveness acceptability curve (CEAC), which demonstrates the probability of an intervention being cost effective at different willingness-to-pay thresholds.

## Results

31 women were randomised into the PLUTO trial before it was stopped early due to difficulties in recruitment. Of those randomised to VAS and conservative management, 3/16 (19%) and 2/15 (13%) respectively did not receive their allocated intervention. Based on intention-to-treat analysis, survival at 28 days was higher if allocated VAS (50%) than conservative management (27%) [RR 1.88 (95% CI 0.71–4.96), P = 0.27)]. At 12 months survival was 44% in the VAS arm and 20% in the conservative arm [RR 2.19 (0.69–6.94), P = 0.25)]. Neither difference was statistically significant.

The results of the economic analysis using intention to treat data are presented in Table 3. For the base case the average cost incurred within the VAS arm was £20,851. The average number of survivors was estimated to be 1.38 at 28 days. The average costs incurred on the conservative arm, on the other hand, was £9,868 and the average number of survivors was estimated to be 0.67. Therefore, the additional cost of VAS was £10,983 for achieving additional 0.71 survivors at 28 days. This translates to an incremental cost effectiveness ratio of £15,506 per survivor at 28 days on the VAS arm. Similarly, the incremental cost per survivor at 1 year can be calculated as £15,415. Therefore, within the ITT analysis, for every additional child that survived up to 28 days (and 1 year), a cost of £15,506 (and £15,415) was incurred. On the other hand, the ICER results for achieving a clinical morbidity free year were substantially higher at £43,932 since very few patients survived to 1 year without any morbidity. ICERs were similarly determined for the per-protocol (PP) and uniform prior (UP) analyses (shown in Tables 3, 4, 5) and these were broadly similar to those derived via ITT.

**Table 3:** Intention to treat analysis – base case, probabilistic and deterministic sensitivity analyses.

Intervention	Total cost (in £)	Survival	Survival	MFS	ICER	ICER	ICER
		(28 days)	(1 yr)	(1 yr)	(28 days)	(1 yr)	(MFS)
**Base case:**							
VAS	20,851	1.38	1.31	0.25	15,506	15,415	43,932
Nil (Conservative)	9,868	0.67	0.6	0			
**Probabilistic sensitivity analysis** :							
VAS	20,901	1.38	1.31	0.25	15,482	15,407	44,145
Nil (Conservative)	9,853	0.67	0.6	0			
**Deterministic analysis 1 (Doubled VAS costs):**							
VAS	21,582	1.38	1.31	0.25	16,382	16,287	46,417
Nil (Conservative)	9,978	0.67	0.6	0			
**Deterministic analysis 2 (Halved VAS costs):**							
VAS	20,492	1.38	1.31	0.25	15,076	14,988	42,715
Nil (Conservative)	9,813	0.67	0.6	0			
**Deterministic analysis 3 (Adjusted outlier costs):**							
VAS	9,212	1.38	1.31	0.25	−926	−920	−2,623
Nil (Conservative)	9,868	0.67	0.6	0			
**Deterministic analysis 4 (as per treatment received):**							
VAS	22,827	2.07	2.00	0.27	8,052	8,071	52,965
Nil (Conservative)	8,703	0.31	0.25	0			

**Table 4:** Per Protocol analysis – base case, probabilistic and deterministic sensitivity analyses.

Intervention	Total cost(in £)	Survival	Survival	MFS	ICER	ICER	ICER
		(28 days)	(1 yr)	(1 yr)	(28 days)	(1 yr)	(MFS)
**Base case:**							
VAS	21,970	1.47	1.4	0.27	15,499	15,272	40,536
Nil (Conservative)	11,161	0.77	0.69	0			
**Probabilistic sensitivity analysis:**							
VAS	21,993	1.47	1.4	0.27	15,510	15,321	40,465
Nil (Conservative)	11,147	0.77	0.69	0			
**Deterministic analysis 1 (Doubled VAS costs):**							
VAS	22,683	1.47	1.4	0.27	16,339	16,103	42,734
Nil (Conservative)	11,287	0.77	0.69	0			
**Deterministic analysis 2 (Halved VAS costs):**							
VAS	21,614	1.47	1.4	0.27	15,080	14,988	42,715
Nil (Conservative)	11,097	0.77	0.69	0			
**Deterministic analysis 3 (Adjusted outlier costs):**							
VAS	9,555	1.47	1.4	0.27	−2,301	−2,268	−6,019
Nil (Conservative)	11,161	0.77	0.69	0			
**Deterministic analysis 4 (as per treatment received):**							
VAS	24,167	2.21	2.14	0.29	7,770	7,770	50,506
Nil (Conservative)	9,736	0.36	0.29	0			

**Table 5:** Uniform Priors analysis – base case, probabilistic and deterministic sensitivity analyses.

Intervention	Total cost(in £)	Survival	Survival	MFS	ICER	ICER	ICER
		(28 days)	(1 yr)	(1 yr)	(28 days)	(1 yr)	(MFS)
**Base case:**							
VAS	20,617	1.29	1.1	0.35	15,327	15,518	31,426
Nil (Conservative)	10,855	0.66	0.47	0.04			
**Probabilistic sensitivity analysis:**							
VAS	21,993	1.29	1.1	0.35	15,364	15,588	31,615
Nil (Conservative)	11,147	0.61	0.42	0.06			
**Deterministic analysis 1 (Doubled VAS costs):**							
VAS	21,316	1.29	1.1	0.35	16,428	16,617	39,330
Nil (Conservative)	10,055	0.61	0.42	0.06			
**Deterministic analysis 2 (Halved VAS costs):**							
VAS	20,268	1.29	1.1	0.35	15,056	15,230	36,041
Nil (Conservative)	9,947	0.61	0.42	0.06			
**Deterministic analysis 3 (Adjusted outlier costs):**							
VAS	8,684	1.29	1.1	0.35	252	255	604
Nil (Conservative)	8,511	0.61	0.42	0.06			
**Deterministic analysis 4 (as per treatment received):**							
VAS	22,094	1.75	1.55	0.37	9,347	9,596	36,917
Nil (Conservative)	9,476	0.4	0.24	0.03			

Doubling (DSA1) or halving (DSA2) the cost of the shunt did not result in a significant change in the ICER values. Thus the results of the base case analysis were not sensitive to changes to VAS cost.

When the high costs incurred by a few of the patients within the trial were substituted with the average cost incurred by all other patients in that arm (DSA3), the overall costs within the VAS arm reduced significantly and the VAS dominated over the conservative management (i.e. VAS demonstrated greater effectiveness at a lower cost compared to the conservative management). Thus whereas some patients receiving VAS were very expensive, others appeared to incur lower costs whilst accruing more benefits.

Two patients who were randomised to the conservative arm received a VAS whilst three patients randomised to the VAS arm did not. When the ITT analysis was repeated as per the treatment received (DSA4), the incremental costs increased slightly (£14,124 vs. £10,983 in base case analysis) while the gains in effectiveness were higher (1.7 vs. 0.71 in base case), resulting in substantially lower ICERs for survival at 28 days and 1 year respectively. The ICER for MFS did not change significantly, however. Similar results were noted with the PP and UP analyses.

**Figure 3:**
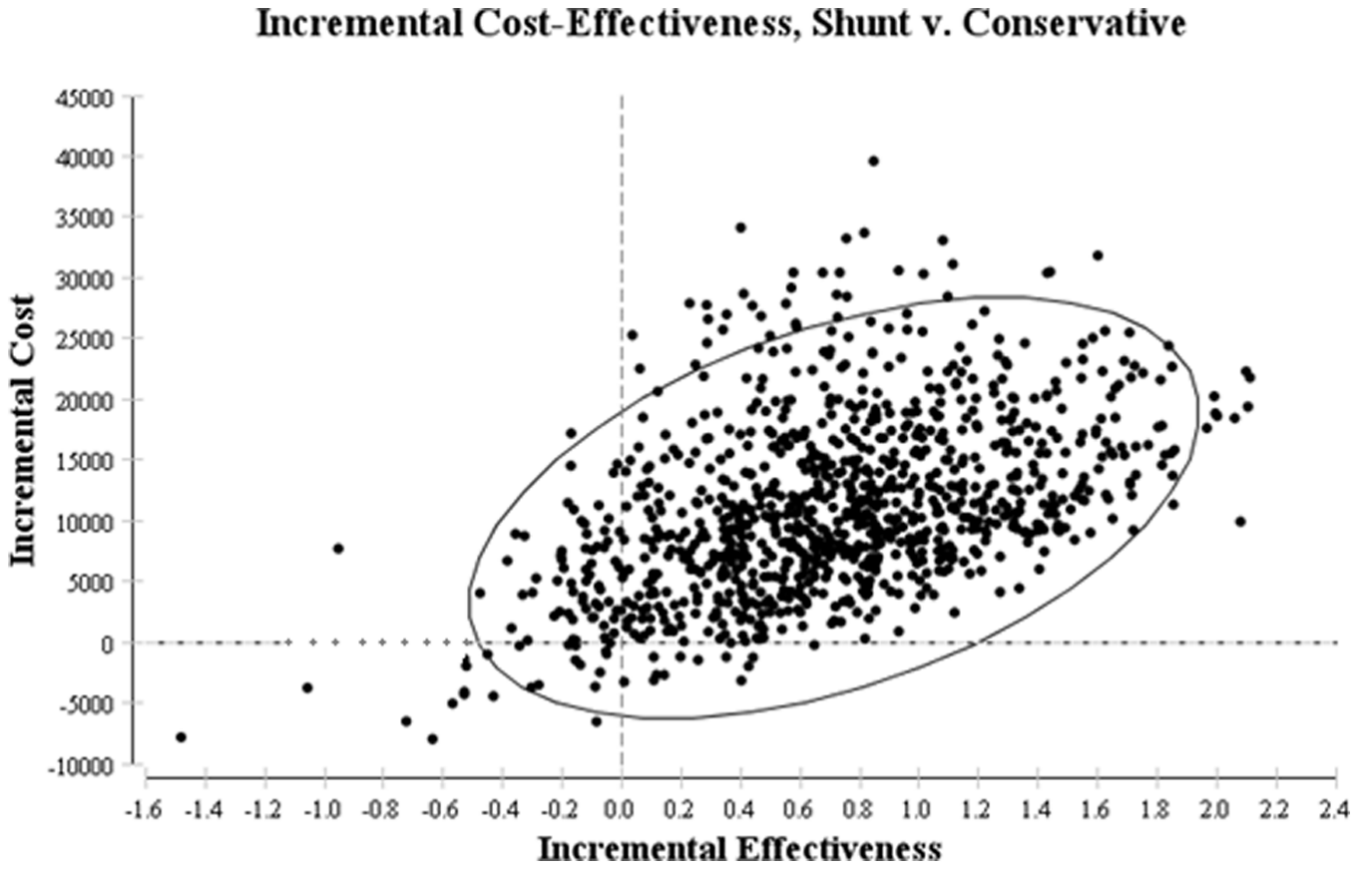
Scatter plot representing Monte Carlo simulation of base case analysis for survival at 28 days. This figure demonstrates the results of the probabilistic sensitivity analysis (PSA) which simultaneously represents uncertainty in cost and effectiveness values. The x and y axes represent the incremental effectiveness and cost of VAS treatment compared with conservative management respectively. For this analysis, 20,000 simulations were carried out. The data points represent the ICER value obtained with each simulation and cumulatively they demonstrate the possible results given the uncertainty in the point estimates for the ICER. The ellipse in this figure represents 95% of all data points, most of which lie in the upper right quadrant. This illustrates that there is a high probability for VAS to be more expensive and more effective (i.e. more likely to result in survival at 28 days) than conservative treatment.

The probabilistic sensitivity analysis results did not show a significant change to the base case ICERs (Tables 3, 4, 5). The scatter plot representing survival at 28 days as per the ITT analysis is shown in figure 3. The X axis in the figure represents difference in effectiveness with VAS use whilst the differences in cost are represented on the Y axis. Most of the data points lie in the right upper quadrant which indicates that VAS placement is likely to be more effective whilst being more expensive for this outcome.

**Figure 4:**
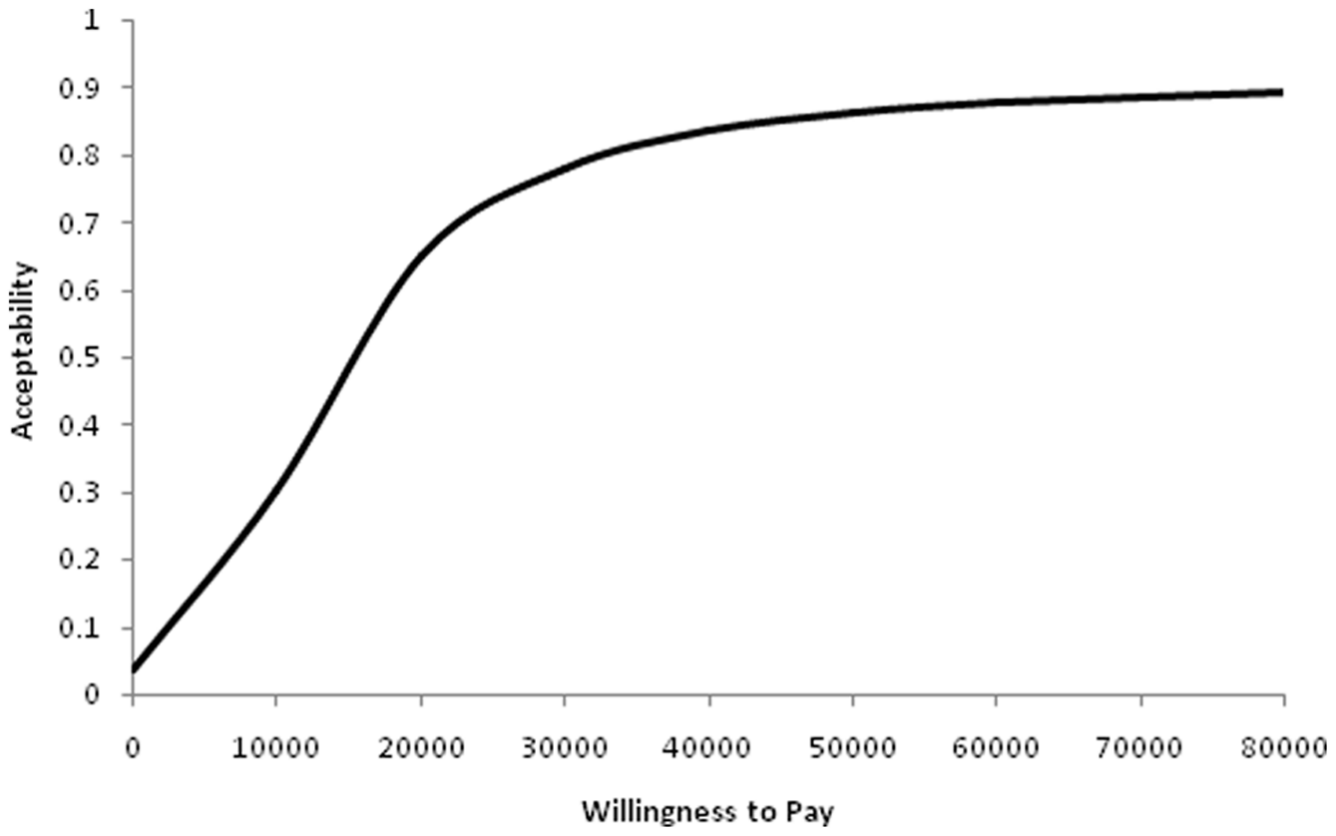
Likelihood of acceptability of VAS at various thresholds of willingness to pay for improving survival at 28 days in affected infants through a cost effectiveness acceptability curve (CEAC). The X axis represents the amounts that the decision maker is willing to pay (WTP) for this outcome and the Y axis represents the likelihood that the intervention will be cost-effective at the given threshold. This graph clearly shows that at lower WTP thresholds, the intervention is unlikely to be cost effective. At higher thresholds, for example at a WTP level of £40,000 per neonate, there is an 80% chance that VAS will be cost effective for achieving survival at 28 days.

Figure 4 represents the cost-effectiveness acceptability curve for survival at 28 days as per the ITT analysis. The curve represents the probability of VAS being cost effective at different willingness to pay thresholds for the procedure. For example, if the decision maker is willing to invest an additional £20,000 for every additional child that survives to 28 days, the probability that VAS is cost-effective is about 65%. However, there is only 23% possibility that a child will survive to one year without morbidity for that amount of investment (data not shown).

## Discussion

### Principal findings

The results from the randomised controlled trial of percutaneous VAS versus conservative treatment are published elsewhere [14]. VAS improved perinatal and 1 year survival for babies with LUTO but it did not improve renal morbidity at 1 year of age in these infants. Indeed, there was very little difference in probability of clinical morbidity free survival for a year (MFS) achieved in the VAS versus conservative arms.

The economic evaluation results reported here show that percutaneous VAS is more expensive than conservative management. For every additional child who survived to 28 days (1 year), the additional cost of opting for VAS over conservative management was £15,506 (£15,415) based on the ITT analysis. The additional cost for obtaining a disease free year with VAS was much higher at £43,932. The results were not altered significantly by changing the cost of VAS. When the analysis was repeated according to treatment received (and not Intention to treat), the costs in the VAS arm reduced relative to base case costs rendering the VAS less expensive for the same survival outcomes.

We also found that some patients who received a VAS utilised disproportionate amount of resources within the first year of life. By substituting these very high costs with the average incurred costs (DSA 3), the cost effectiveness of VAS was significantly improved, even conferring dominance (i.e., providing higher benefit at a lower cost than the conservative option). Although, for the purposes of this analysis, we assumed that the high cost incurred by a few patients in this trial was entirely coincidental which is unlikely to be the case. It is remarkable that all the patients with high overall costs (greater than £50,000) were within the VAS group. The high costs incurred were almost entirely due to the prolonged NICU admission immediately after birth in these infants (data not shown). It is possible that these infants would have succumbed to LUTO in the absence of VAS (the infants with “poor prognosis” on antenatal scans [15]). These children continued to have higher admission rates in the first year (beyond the neonatal period) and are responsible for the higher costs in VAS arm relative to the conservative arm. It should be noted that these patients had previously been thought to benefit the most from VAS placement [5], [16]. Although survival in these infants appears to improve with VAS, the PLUTO trial has clearly demonstrated that they suffer with significant morbidities leading to higher healthcare costs.

Presenting results in terms of cost per QALY enables comparison of costs across disease areas and is considered the corner stone for health policy decisions within the UK. However, as discussed previously, QALYs or other utility measures could not be obtained in this trial. Nevertheless we could extrapolate the ICER results obtained to surmise that an extra 0.77 QALYs (i.e. more than 9 months in full health) will need to be obtained in the VAS arm compared with the conservative arm to make the intervention cost effective at 1 yr (assuming a cost utility threshold of £20,000 per QALY). From the data obtained from this trial and from other studies, it appears unlikely that VAS may result in such significant QALY gains in these infants. Hence, it is safe to conclude that VAS is not likely to be cost effective in the management of these patients.

### Strengths and limitations of the study

The main strength of this economic analysis is that it is the first based on a randomised trial in this clinical area. The data is directly derived from the PLUTO study which is the largest randomised controlled trial for LUTO to date (albeit small in absolute terms). This analysis benefits from collaborative work between clinicians, nurses, statisticians and health economists. Furthermore, resource use data were collected prospectively within the trial and accurately represent actual UK NHS costs incurred by the participants.

One major limitation of the study was the small sample size which introduces uncertainties into the analysis. However, given that LUTO is an uncommon condition, it may not be possible to carry out larger studies in this disease area in the future. Furthermore, the model based approach facilitated the inclusion of uncertainty into the analysis and this was explicitly incorporated into the results. We carried out three different analyses for the base case and have also incorporated extensive deterministic and probabilistic sensitivity analyses.

Another limitation is that these findings only apply to those babies who were included into the randomised arm of the PLUTO trial. All those babies where the clinicians felt sure of the advantages (or futility) of VAS were included into the registry arm of the trial. It was felt that inclusion of these patients would introduce bias into the analysis and was hence not considered.

For the purpose of this analysis, we assumed that all centres had similar expertise. However, for any given primary surgical trial, different levels of experience and expertise may be encountered at the different recruiting centres. These differences could not be explored within this analysis given the small patient numbers.

It can be argued that the longer term effects of LUTO (e.g. cognition, continence, infertility, and need for dialysis or renal transplantation) will have a bearing on the overall effectiveness of VAS in the management of these patients. Indeed, the actual costs of renal morbidity do not become apparent until the child is over a year of age since dialysis is not favoured in infants. Resource use data from the longer term management of these children will, therefore, be important in determining the cost effectiveness of the intervention. Reliable data regarding long term effects of LUTO are not available in the literature and hence we did not extend the model beyond one year. However, longer term follow up of the PLUTO participants is planned and a further analysis of these data may be possible in the future.

### Strengths and limitations in relation to other studies

Despite the difficulties with recruitment, PLUTO is the largest randomised controlled trial looking at the effectiveness of VAS for management of LUTO and the first to prospectively estimate the cost-effectiveness of the intervention.

Other researchers who studied these patients prospectively for a longer period have reported significant renal morbidity (notably end stage renal failure requiring dialysis or transplantation) [17] and few others have found acceptable QoL scores in a majority of their patients in the long term [18]. Such data, which will be useful in understanding the overall cost-effectiveness of VAS, are not currently available from the PLUTO trial.

### Meaning of this study

This analysis demonstrated that while a VAS may improve survival, it is a more expensive option than conservative management in patients with LUTO.

Most infants who survived to 28 days managed to survive to 1 year in this study. Using survival alone as a marker of efficacy in these patients can be misleading however, since most of the survivors are left with significant renal morbidity. Therefore, the effectiveness of the intervention can be overstated if only survival is considered in the analysis. On the other hand, a small cross-sectional study of children aged 1–14 yrs (mean 5.83 yrs) with LUTO who received intrauterine VAS reported Quality of Life (QoL) scores comparable to those of the general healthy population [18]. Thus longer term follow-up of patients recruited into the PLUTO trial may provide greater clarity regarding the clinical and cost effectiveness of VAS in LUTO.

### Conclusion

We conclude that whilst VAS may improve neonatal survival in LUTO, it is more expensive compared to the conservative approach. Furthermore, it does not result in significant improvements in morbidity and is therefore not likely to be cost effective in the management of these patients given the NICE cost threshold of £20,000 per QALY.

### Unanswered questions and future research

In order to obtain a better understanding regarding the outcomes of VAS in LUTO, a larger study needs to be carried out. However, this may be difficult given the infrequent occurrence of this condition as well as the problems with recruitment that have previously been discussed. Within the PLUTO trial, longer term follow up of patients in the registry as well as the randomisation arm is planned and this may further clarify questions regarding effectiveness of VAS in LUTO.

## Supporting Information

File S1 **Table S1 Resource use data**. Details of resource use obtained directly from the clinical trial are shown. **Table S2 Delivery Costs.** Cost of delivery within the UK NHS (which were used in the cost estimation for the trial) are listed along with the appropriate Healthcare Resource Group (HRG) codes used to obtain these costs. **Table S3** **Admission and Transportation costs**. Cost inputs for neonatal admission within the trial were estimated from published UK NHS tariffs. These are shown alongside the relevant HRG codes. **Table S4 Cost of procedures.** Costs of all the procedures carried out during the trial using the UK NHS tariffs are shown. HRG codes are also provided. **Table S5 Cost of investigations.** Costs of investigations used in the analysis are listed and the sources are made explicit. **Table S6 Model inputs for the conservative arm of the trial.** The inputs used for all three base case analyses (intention to treat, per protocol and uniform prior analyses) are shown. The inputs were derived from trial data. **Table S7 Model inputs for the patients who had Vesico-Amniotic Shunt (VAS) placed and developed complications within the trial**. All three base case analyses inputs are shown. **Table S8 Model inputs for patients who had VAS placed but did not have complications**. All base case analyses inputs are shown. **Supplementary text:** Details of the per protocol and uniform prior analyses are discussed.
(DOCX)
