# Supplementary material for: Evaluation of the Cost Effectiveness of Vesico-Amniotic Shunting in the Management of Congenital Lower Urinary Tract Obstruction (Based on Data from the PLUTO Trial)
Source: PLoS One. 2013 Dec 20;8(12):e82564. doi: 10.1371/journal.pone.0082564 (PMC3869698; doi:10.1371/journal.pone.0082564)
Supplement: File S1 — Table S1 Resource use data. Details of resource use obtained directly from the clinical trial are shown. Table S2 Delivery Costs. Cost of delivery within the UK NHS (which were used in the cost estimation for the trial) are listed along with the appropriate Healthcare Resource Group (HRG) codes used to obtain these costs. Table S3 Admission and Transportation costs. Cost inputs for neonatal admission within the trial were estimated from published UK NHS tariffs. These are shown alongside the relevant HRG codes. Table S4 Cost of procedures. Costs of all the procedures carried out during the trial using the UK NHS tariffs are shown. HRG codes are also provided. Table S5 Cost of investigations. Costs of investigations used in the analysis are listed and the sources are made explicit. Table S6 Model inputs for the conservative arm of the trial. The inputs used for all three base case analyses (intention to treat, per protocol and uniform prior analyses) are shown. The inputs were derived from trial data. Table S7 Model inputs for the patients who had Vesico-Amniotic Shunt (VAS) placed and developed complications within the trial. All three base case analyses inputs are shown. Table S8 Model inputs for patients who had VAS placed but did not have complications. All base case analyses inputs are shown. Supplementary text: Details of the per protocol and uniform prior analyses are discussed. (DOCX) [file pone.0082564.s001.docx]

| **Resource** | **VAS*(n=4)** | **VAS**(n=12)** | **Conservative(n=15)** |
| --- | --- | --- | --- |
| Antenatal U/S scan | 1.5 | 6.5 | 4.4 |
| VAS | 1.25 | 1.25 | 0.1333 |
| Karyotype testing | 0 | 0.25 | 0.33 |
| Antenatal Urinalysis | 0.25 | 0.5 | 0 |
| Post mortem examination | 0 | 0.083 | 0.27 |
| Delivery: |  |  |  |
| Normal vaginal | 0.75 | 0.58 | 0.47 |
| Vaginal breech | 0 | 0 | 0.067 |
| Vaginal ToP | 0.25 | 0.083 | 0.133 |
| Caesarean section | 0 | 0.33 | 0.33 |
| Admission: (up to age 60 days) | 15 | 12.67 | 8.4 |
| NICU | 15 | 11.5 | 4 |
| HDU | 0 | 0.17 | 0 |
| Ward | 0 | 1 | 4.4 |
| Surgeries: |  |  |  |
| CR^ | 0 | 0.17 | 0.067 |
| Shunt removal | 0 | 0 | 0.067 |
| Vesicostomy | 0.25 | 0.17 | 0.067 |
| Nephrostomy | 0.25 | 0.083 | 0 |
| Procedures: |  |  |  |
| MCUG | 0.25 | 0 | 0.067 |
| Cystoscopy | 0.5 | 0.17 | 0 |
| Video urodynamic study | 0 | 0 | 0.067 |
| other | 1.25^$^ | 0.42^$$^ | 0 |
| Peritoneal dialysis (person-yrs) | 0 | 0.028 | 0 |
| Laboratory tests: |  |  |  |
| Serum Creatinine | 0 | 0.5 | 0.2 |
| Sodium | 0.25 | 0 | 0.067 |
| Urinary Sodium | 0 | 0 | 0.067 |
| Urinary Calcium | 0.25 | 0 | 0.067 |
| Creatinine Clearance | 0 | 0.083 | 0.067 |
| Other: |  |  |  |
| Renal Ultrasound Scan | 0.25 | 0.5 | 0.29 |
| Admission (age 60 days-1 year) | 25.5 | 11.1 | 4.07 |

Table S1: Resource use data

*Shunt arm with complications **Shunt arm without complications ^Cystoscopic resection of the posterior urethral valve$Other procedures were insertion of Hickman line ,removal of suprapubic catheter, re-insertion of suprapubic catheter (x3); $$Other procedures included peritoneal dialysis catheter insertion, DMSA scan, MAG-3 scan, circumcision

Table S2: Delivery costs*:

| **Description** | **Unit Cost (£)** | **Source** |
| --- | --- | --- |
| Normal Vaginal delivery without cc^^^ | £1,236 | Normal delivery no cc (NZ01F)^#^ |
|  |  |  |
| Normal Vaginal delivery with cc | £1,906 | Normal delivery with cc (NZ01E)^#^ |
|  |  |  |
| Elective Caesarean section | £2,378 | Caesarean section no cc (NZ03D)^#^ |
|  |  |  |
| Emergency Caesarean section | £3,236 | Caesarean section with cc (NZ03C)^#^ |
| Medical termination of pregnancy | £431 | (MA18Z)^#^ |
| *All costs assumed for women aged between 16 and 40 yrs of age# Obtained from NHS reference costs 2011-12  ^ cc stands for complications | | |

Table S3:Admission and transportation costs:

| **Description** | **Cost (£)** | **Source** |
| --- | --- | --- |
| Neonatal Intensive Care (ICU) | £1117**^^^** | PbR 2008-9* cost code:XA01Z |
|  |  |  |
| High Dependency Unit (HDU) | £785**^^^** | PbR 2008-9* cost code:XA02Z |
|  |  |  |
| Special care (SCBU) | £490**^^^** | PbR 2008-9* cost code:XA03Z |
|  |  |  |
| Neonatal care transportation | £857 | PbR 2008-9* cost code:XA06Z |
|  |  |  |
| Neonatal critical care normal care | £460**^^^** | PbR 2008-9* cost code:XA05Z |
| * costs inflated to 2010-11 prices using the PSSRU inflation indices  ^ cost per day | | |

Table S4: Costs of Procedures:

| **Description** | **Cost (£)** | **Source** |
| --- | --- | --- |
| Cystoscopic resection of PUV | £1,380 | Bladder major procedure (LB13B)* |
|  |  |  |
| Nephrectomy | £4,430 | Kidney major open procedure (LB02D)* |
|  |  |  |
| Nephrostomy | £2,282 | Percutaneous nephrostomy no cc (LB01B)* |
|  |  |  |
| Vesicostomy | £803 | Bladder intermediate procedure (LB14D)* |
|  |  |  |
| Vesicostomy closure | £703 | Bladder minor procedure (LB15D)* |
|  |  |  |
| Peritoneal dialysis catheter insertion | £1,508 | Peritoneal dialysis related procedure, no cc (LA05B)* |
|  |  |  |
| Orchipexy | £1,105 | Testes open procedure (LB34C)* |
|  |  |  |
| Circumcision | £740 | Penile minor procedure (LB32C)* |
|  |  |  |
| Cystoscopy | £803 | Bladder intermediate procedure (LB14D)* |
|  |  |  |
| Suprapubic catheter insertion (and replacement) | £703 | Bladder minor procedure (LB15D)* |
|  |  |  |
| Shunt removal | £703 | Bladder minor procedure (LB15D)* |
|  |  |  |
| Amnioinfusions | £445 | Detailed scan including counseling time (source:BWH) |
|  |  |  |
| Micturatingcystourethrogram | £340 | Dynamic studies of the urinary tract (LB42Z)* |
|  |  |  |
| Peritoneal dialysis | £67 | Cost per day of continuous ambulatory peritoneal dialysis for children 2008/9 (RD3C), inflated to 2010-11 prices |
| *Obtained from NHS reference costs 2011-12 | | |

Table S5: Cost of investigations:

| **Description** | **Cost (£)** | **Source** |
| --- | --- | --- |
| Serum creatinine | £3 | Local laboratory (BHH)* |
|  |  |  |
| Creatinine Clearance | £5 | Local laboratory (BHH)* |
|  |  |  |
| B_2_-microglobin | £3 | Local laboratory (BHH)* |
|  |  |  |
| Urea and Electrolytes | £3 | Local laboratory (BHH)* |
|  |  |  |
| Urinary Calcium | £4 | Local laboratory (BHH)* |
|  |  |  |
| Urinary Sodium | £4 | Local laboratory (BHH)* |
|  |  |  |
| Full blood count | £2 | Local laboratory (BHH)* |
|  |  |  |
| Karyotyping | £197 | Q-F PCR trisomy test + sex chromosome (BWH)^#^ |
|  |  |  |
| Post mortem examination | £1440 | From Roberts,TE et al(Roberts 1998) inflated to 2010/11 prices |
| *BHH: Birmingham Heartlands Hospital  #BWH: Birmingham Women’s Hospital | | |

**Table S6: Conservative arm [probability (number of patients, total number in each branch**

**of the tree)]**

| **Parameter (distribution)** | **ITT^*^** | **PP^#^** | **UP^^^** |
| --- | --- | --- | --- |
| Pregnancy lost < 24 weeks (Beta) | 0.2 (3,15) | 0.08 (1,13) | 0.24 (4,17) |
| - Chorioamnionitis (Dirichlet) | 0 (0,3) | 0 (0,0) | 0.17 (1,6) |
| - Termination (Dirichlet) | 0.67 (2,3) | 0 (0,0) | 0.5 (3,6) |
| - Miscarriage (Dirichlet) | 0.33 (1,3) | 1 (1,1) | 0.33 (2,6) |
| Pregnancy continued > 24 weeks (Beta) | 0.8 (12,15) | 0.92 (12,13) | 0.76 (13,17) |
| Died < 28 days after birth (Beta) | 0.67 (8,12) | 0.67 (8,12) | 0.64 (9,14) |
| Died in utero > 24 weeks (Dirichlet) | 0 (0,8) | 0 (0,8) | 0.09 (5,14) |
| - Chorioamnionitis (Beta) | 0 (0,0) | 0 (0,0) | 0.5 (1,2) |
| - Other (Beta) | 0 (0,0) | 0 (0,0) | 0.5 (1,2) |
| - Died at delivery (Dirichlet) | 0.75 (6,8) | 0.75 (6,8) | 0.64 (7,11) |
| - Neonatal death (Dirichlet) | 0.25 (2,8) | 0.25 (2,8) | 0.27 (3,11) |
| Survived >28days after birth | 0.33 (4,12) | 0.33 (4,12) | 0.36 (5,14) |
| - Died before 1yr (Beta) | 0.25 (1,4) | 0.25 (1,4) | 0.33 (2,6) |
| - Survived at 1 yr (Beta) | 0.75 (3,4) | 0.75 (3,4) | 0.67 (4,6) |
| - No morbidity (Beta) | 0 (0,3) | 0 (0,3) | 0.2 (1,5) |
| Renal impairment | 1 (3,3) | 1 (3,3) | 0.8 (4,5) |
| - Mild impairment (Dirichlet) | 0 (0,3) | 0 (0,3) | 0.17 (1,6) |
| - Moderate impairment (Dirichlet) | 1 (3,3) | 1 (3,3) | 0.67 (4,6) |
| - Severe impairment (Dirichlet) | 0 (0,3) | 0 (0,3) | 0.17 (1,6) |

Intention to Treat analysis # Per protocol analysis ^ Uniform prior analysis ● represents terminal nodes

Note: ITT and per protocol costs were obtained from the PLUTO trial; For the uniform prior analysis we assumed equal pre-trail probabilities for all the pathways within the model and used ITT values from the trial to obtain posterior probabilities.

**Table S7: VAS arm (with complications)** **[probability (number of patients, total number in each branch of the tree)]**

| **Parameter (distribution)** | **ITT^*^(n=16)** | **PP^#^(n=15)** | **UP^^^** |
| --- | --- | --- | --- |
| Complications (Beta) | 0.25 (4,16) | 0.27 (4,15) | 0.28 (5,18) |
| Pregnancy lost < 24 weeks (Beta) | 0.75 (3,4) | 0.75 (3,4) | 0.67 (4,6) |
| Chorioamnionitis (Dirichlet) | 0.67 (2,3) | 0.67 (2,3) | 0.5 (3,6) |
| Termination (Dirichlet) | 0 (0,3) | 0 (0,3) | 0.17 (1,6) |
| Miscarriage (Dirichlet) | 0.33 (1,3) | 0.33 (1,3) | 0.33 (2,6) |
| Pregnancy continued > 24 weeks (Beta) | 0.25 (1,4) | 0.25 (1,4) | 0.33 (2,6) |
| Died < 28 days after birth (Beta) | 0 (0,1) | 0 (0,1) | 0.33 (1,3) |
| Died in utero > 24 weeks (Dirichlet) | 0 (0,0) | 0 (0,0) | 0.33 (1,3) |
| Chorioamnionitis (Beta) | 0 (0,0) | 0 (0,0) | 0.5 (1,2) |
| Other (Beta) | 0 (0,0) | 0 (0,0) | 0.5 (1,2) |
| Died at delivery (Dirichlet) | 0 (0,0) | 0 (0,0) | 0.33 (1,3) |
| Neonatal death (Dirichlet) | 0 (0,0) | 0 (0,0) | 0.33 (1,3) |
| Survived >28days after birth | 1 (1,1) | 1 (1,1) | 0.67 (2,3) |
| - Died before 1yr (Beta) | 0 (0,1) | 0 (0,1) | 0.33 (1,3) |
| - Survived at 1 yr (Beta) | 1 (1,1) | 1 (1,1) | 0.67 (2,3) |
| - No morbidity (Beta) | 0 (0,1) | 0 (0,1) | 0.33 (1,3) |
| Renal impairment | 1 (1,1) | 1 (1,1) | 0.67 (2,3) |
| - Mild impairment (Dirichlet) | 1 (1,1) | 1 (1,1) | 0.5 (2,4) |
| - Moderate impairment (Dirichlet) | 0 (0,1) | 0 (0,1) | 0.25 (1,4) |
| - Severe impairment (Dirichlet) | 0 (0,1) | 0 (0,1) | 0.25 (1,4) |
| * Intention to Treat analysis # Per protocol analysis ^ Uniform prior analysis ● represents terminal nodes  Note: ITT and per protocol costs were obtained from the PLUTO trial; For the uniform prior analysis we assumed equal pre-trail probabilities for all the pathways within the model and used ITT values from the trial to obtain posterior probabilities. | | | |

**Table S8: VAS arm (no complications): [probability (number of patients, total number in each branch of the tree)]**

| **Parameter (distribution)** | **ITT^*^(n=16)** | **PP^#^(n=15)** | **UP^^^** |
| --- | --- | --- | --- |
| No complications (Beta) | 0.75 (12,16) | 0.73 (11,15) | 0.72 (13,18) |
| Pregnancy lost < 24 weeks (Beta) | 0.08 (1,12) | 0 (0,11) | 0.14 (2,14) |
| - Termination (Beta) | 1 (1,1) | 0 (0,0) | 0.67 (2,3) |
| - Miscarriage (Beta) | 0 (0,1) | 0 (0,0) | 0.33 (1,3) |
| Pregnancy continued > 24 weeks (Beta) | 0.92 (11,12) | 1 (11,11) | 0.86 (12,14) |
| Died < 28 days after birth (Beta) | 0.36 (4,11) | 0.36 (4,11) | 0.38 (5,13) |
| Died in utero > 24 weeks (Dirichlet) | 0 (0,4) | 0 (0,4) | 0.14 (1,7) |
| - Pregnancy loss (Beta) | 0 (0,0) | 0 (0,0) | 0.5 (1,2) |
| - Other (Beta) | 0 (0,0) | 0 (0,0) | 0.5 (1,2) |
| - Died at delivery (Dirichlet) | 1 (4,4) | 1 (4,4) | 0.71 (5,7) |
| - Neonatal death (Dirichlet) | 0 (0,4) | 0 (0,4) | 0.14 (1,7) |
| Survived >28days after birth | 0.64 (7,11) | 0.64 (7,11) | 0.61 (8,13) |
| - Died before 1yr (Beta) | 0.14 (1,7) | 0.14 (1,7) | 0.22 (2,9) |
| - Survived at 1 yr (Beta) | 0.86 (6,7) | 0.86 (6,7) | 0.78 (7,9) |
| - No morbidity (Beta) | 0.33 (2,6) | 0.33 (2,6) | 0.38 (3,8) |
| Renal impairment | .67 (4,6) | .67 (4,6) | 0.62 (5,8) |
| - Mild impairment (Dirichlet) | 0 (0,4) | 0 (0,4) | 0.14 (1,7) |
| - Moderate impairment (Dirichlet) | 1 (4,4) | 1 (4,4) | 0.72 (5,7) |
| - Severe impairment (Dirichlet) | 0 (0,4) | 0 (0,4) | 0.14 (1,7) |
| * Intention to Treat analysis # Per protocol analysis ^ Uniform prior analysis ● represents terminal nodes  Note: ITT and per protocol costs were obtained from the PLUTO trial; For the uniform prior analysis we assumed equal pre-trail probabilities for all the pathways within the model and used ITT values from the trial to obtain posterior probabilities. | | | |

**Base case analyses:**

In the Intention to treat (ITT) method, patients are “compared in the group to which they were initially randomised”(Sally and Fiona 1999). This enables estimation of benefit in a real life situation following policy change rather than the hypothetical situation when treatment is provided exactly as planned. Not using ITT analysis can often exaggerate the benefits of a given intervention and it is widely acknowledged to be the least biased of analysis options(Begg, Cho et al. 1996) .

However, given the small numbers of patients involved in the RCT, we carried out three separate base case analyses to explore data uncertainty. These included the per-protocol and uniform prior analyses in addition to the ITT analysis (which has been discussed in detail in the main manuscript).

The latter two analyses are discussed in detail in this section.

Per protocol analysis :

Within the trial, three expectant mothers chose to terminate the pregnancy after they were randomised into the trial. This represented about 10% of all patients randomised into the trial and therefore, there was a possibility that this could significantly impact on the findings of the economic analysis. In order to consider this issue, a separate per protocol analysis was carried out excluding these patients within the trial.

Thus one woman from the VAS arm and two women from the conservative arm of the trial were excluded bringing the total number of individuals captured within the analysis to 28. It should be noted that protocol violation due to incorrect treatment allocation (i.e. those patients who were randomised into conservative arm but received a VAS and vice-versa) is not considered in this analysis but is addressed in a separate deterministic sensitivity analysis.

Uniform Prior analysis :

Given the small numbers of patients recruited into the trial, the outcomes and costs noticed in the trial may not be truly representative of real life scenarios. This was especially true where there were no data available for some of the pathways created within the decision tree model.

Since PLUTO is the largest clinical trial for this condition to date, it was not possible to obtain other systematically collected data regarding this rare condition. We therefore carried out a pragmatic analysis employing Bayesian principles. In this analysis, we assumed that all patients had uniform prior possibility of going down any of the designated branches from a given node. To these uniform priors, we added the trial data to obtain posterior probabilities which were inputted into the decision tree.

For example, in the conservative arm, the data for the three possibilities following pregnancy loss at less than 24 weeks were 0 cases of chorioamnionitis, 2 cases of termination, 1 case of miscarriage in the ITT dataset. Assuming a uniform prior (i.e. an equal prior value of 1 applied to each arm of the tree), the posterior distribution is equivalent to 1 case of chorioamnionitis, 3 cases of termination, 2 cases of miscarriage. These three possibilities were now assigned a Dirichlet distribution within the model. This method has been previously suggested by Briggs et al(Briggs A, Claxton K et al. 2006).

Resource use data obtained from the trial are shown in table S1. Cost data were derived from various sources as shown in tables S2-S5. Clinical data inputs into the model for these analyses were derived from the PLUTO trial and are shown in tables S6 –S8. Since LUTO is a rare condition and since the pregnancies associated with this condition cannot be considered ‘normal’, normal pathways of care cannot apply to these patients. The closest group to the hypothetical cohort of babies that were considered in the UP analysis, therefore, would be those included in the trial. The cost data for the uniform prior (UP) analysis were, therefore, derived from the trial.

Assumptions:

In order to calculate costs for the uniform prior analysis, a few further assumptions were made:

- Apart from costs arising directly from complications, the costs incurred in the VAS arm with and without complications were assumed to be the same. For example, costs incurred by a patient with moderate renal failure on the VAS with no complications arm was assumed to be similar to that of patient on the VAS with complications arm except for costs directly incurred due to complications.
- Prenatal costs incurred in the VAS and conservative arms were assumed to be similar (apart from direct costs due to VAS placement and complications, if any). Differences in outcomes (and hence in costs) were considered only after birth.

As with the intention to treat analysis, deterministic and probabilistic sensitivity analyses were carried out within these methods to further explore data uncertainty.

Results and conclusion:

As shown in tables 4 and 5 in the main manuscript, the incremental cost effectiveness ratios (ICERs) derived from the per-protocol analysis are quite similar to those obtained using the uniform priors method. These, in turn, are similar to those derived from the ITT data (table 3 within the main manuscript).

Thus, these analyses also support the ITT conclusion that VAS is more expensive than conservative management in these patients.

**Reference:**

Begg, C., M. Cho, et al. (1996). "Improving the quality of reporting of randomized controlled trials. The CONSORT statement." JAMA **276**(8): 637-9.

Briggs A, Claxton K, et al. (2006). *Decision Modelling for Health Economic Evaluation*. Oxford, Oxford University Press.

Roberts, T. E. (1998). "Economic evaluation and randomised controlled trial of extracorporeal membrane oxygenation: UK collaborative trial. The Extracorporeal Membrane Oxygenation Economics Working Group." BMJ **317**(7163): 911-5; discussion 915-6.

Sally, H. and C. Fiona (1999). "What is meant by intention to treat analysis? Survey of published randomised controlled trials." BMJ **319**(7211): 670-674.
